# Compressing Networks with Super Nodes

**DOI:** 10.1038/s41598-018-29174-3

**Published:** 2018-07-18

**Authors:** Natalie Stanley, Roland Kwitt, Marc Niethammer, Peter J. Mucha

**Affiliations:** 1 Curriculum in Bioinformatics and Computational Biology, University of North Carolina at Chapel Hill, North Carolina, USA; 2 Department of Computer Science, University of Salzburg, Salzburg, Austria; 3 Department of Computer Science, University of North Carolina at Chapel Hill, North Carolina, USA; 4 Carolina Center for Interdisciplinary Applied Mathematics, University of North Carolina at Chapel Hill, North Carolina, USA

## Abstract

Community detection is a commonly used technique for identifying groups in a network based on similarities in connectivity patterns. To facilitate community detection in large networks, we recast the network as a smaller network of ‘super nodes’, where each super node comprises one or more nodes of the original network. We can then use this super node representation as the input into standard community detection algorithms. To define the seeds, or centers, of our super nodes, we apply the ‘CoreHD’ ranking, a technique applied in network dismantling and decycling problems. We test our approach through the analysis of two common methods for community detection: modularity maximization with the Louvain algorithm and maximum likelihood optimization for fitting a stochastic block model. Our results highlight that applying community detection to the compressed network of super nodes is significantly faster while successfully producing partitions that are more aligned with the local network connectivity and more stable across multiple (stochastic) runs within and between community detection algorithms, yet still overlap well with the results obtained using the full network.

## Introduction

Networks appear across disciplines as natural data structures for modeling relational definitions between entities, such as regulatory interactions between genes and proteins, and social connections between people. In practice, most networks are large and have intricate substructures that can be interrogated for further insights about the underlying data. A common practice for extracting subgraphs of interest is community detection^1–3^, which aims to partition network nodes into groups based on the group-level connectivity patterns. For example, assortative communities are typically defined through some measure of a greater weight of within-group edges compared to those between groups. Communities can be identified through various optimization problems, including likelihood maximization^4–6^ and quality function optimization^7–9^, or from spectral properties of associated matrices^10^. In this paper, we seek to explore whether a compressed, smaller version of the network can be used as input to community detection algorithms and produce a partition of nodes in the network that closely resembles the result that would have been obtained using the full network.

Much of our motivation for this network pre-processing step for community detection is inspired by the image analysis literature. The identification of communities in networks is in some ways similar to multi-label image segmentation, which aims to partition a grid of pixels into contiguous regions corresponding to objects in the image. In this sense, each segmented region can be viewed as a community^11^. To speed up segmentation for large images, a popular approach is to avoid computing segmentations at the pixel level and instead reformulate the segmentation problem based on larger-scale image primitives that are likely part of the same partition. Specifically, this can be accomplished by *super pixels* that aggregate pixels together in a way that faithfully adheres to image boundaries, maintaining or improving segmentation accuracy^12^. The SLIC super pixel method^12^ chooses seed pixels across the image’s pixel grid to serve as the super pixel centers and then iteratively grows out and recomputes based on aggregation with neighboring pixels with similar visual features.

When defining super pixels, various authors have typically based the quality of their super pixel representation of the original image on two criteria. First, they seek to minimize *under segmentation error*^13^, which quantifies the extent to which the super pixels bleed across original boundaries in the image. Second, they consider the correctness of the segmentation when applied to the super pixel representation of the image in terms of what would have been obtained using all pixels individually. For the super node network analog, we seek to define super nodes that also minimize under segmentation error and produce communities with high similarity to the result obtained using the full network.

## Problem Formulation

For a network with *N* nodes that we will split into *K* communities, we seek to find a representation of the network with *S* super nodes optimizing the following two quantities. First, given the set **s** = {*s*_1_, *s*_2_, … *s*_*S*_} of *S* super nodes and *K* communities, **k** = {*k*_1_, *k*_2_, … *k*_*K*_} identified with the full network, we wish to minimize the under segmentation error,

1 $$U=\frac{1}{K}\,\sum _{{k}_{i}=1:K}\,\frac{[{\sum }_{{s}_{j}|{s}_{j}\cap {k}_{i}\ne \varnothing }\,|{s}_{j}|]-|{k}_{i}|}{|{k}_{i}|},$$

where $|\cdot |$ represents the count or number of nodes in the indicated set.

We let **z**^Full^ and **z**^SN^ denote the node-to-community assignments for the full network and super node network representations, respectively. To compute the similarity between **z**^Full^ and **z**^SN^, we use Normalized Mutual Information (NMI)^14^. That is, for partitions **z**^Full^ and **z**^SN^ with *p* and *q* communities, respectively, with *N* the *R* × *C* contingency table matrix where *N*_*ij*_ gives the count of the number of shared nodes in community *i* in **z**^Full^ and community *j* in **z**^SN^, the NMI between the two partitions is

2 $${\rm{NMI}}({{\bf{z}}}^{Full},{{\bf{z}}}^{SN})=\frac{-2\,{\sum }_{i}\,{\sum }_{j}\,{N}_{ij}\,\mathrm{log}\,\frac{{N}_{ij}N}{{N}_{i.}{N}_{j.}}}{{\sum }_{i}\,{N}_{i.}\,\mathrm{log}\,\frac{{N}_{i.}}{N}+{\sum }_{j}\,{N}_{.j}\,\mathrm{log}\,\frac{{N}_{.j}}{N}},$$

where *N*_*i*._ and *N*_.*j*_ are the marginal sums over the corresponding rows and columns and $N={\sum }_{i}\,{N}_{i.}={\sum }_{j}\,{N}_{.j}={\sum }_{ij}\,{N}_{ij}$.

## Using Super Nodes In Community Detection

While there are a variety of approaches to identify communities, in this paper we specifically examine how the compressed version of a network can be used in modularity maximization and likelihood maximization. We denote the network adjacency matrix by **A**, where *a*_*ij*_ encodes the edge between nodes *i* and *j*. To maximize modularity in a network with *N* nodes, one seeks to find the node-to-community assignment vector **z** = [*z*_1_, *z*_2_, … *z*_*N*_] that maximizes modularity, *Q*, defined by

3 $$Q=\frac{1}{2M}\,\sum _{i,j}\,[{a}_{ij}-\gamma \frac{{d}_{i}{d}_{j}}{2M}]\,\delta ({z}_{i},{z}_{j}),$$

where *a*_*ij*_ are the adjacency matrix elements encoding presence (and possibly weight) of a possible edge between nodes *i* and *j*, ${d}_{i}={\sum }_{j}\,{a}_{ij}$ gives the strength of node *i*, *δ*(*z*_*i*_, *z*_*j*_) = 1 if nodes *i* and *j* have the same community assignment and 0 otherwise, *M* denotes the number of edges and *γ* is the resolution parameter controlling community sizes. The Louvain algorithm^7^ is a state-of-the-art heuristic for modularity maximization in terms of computational complexity and efficiency. It is an agglomerative method that begins with each node in its own community and merges together nodes and groups of nodes in each agglomeration step to best increase modularity at each level of agglomeration.

An alternative, statistical approach to identify community structure can be obtained through likelihood maximization by fitting a stochastic block model (SBM). Such models assume that the connectivity patterns between the *N* nodes in a network with *K* communities can be modeled according to their community memberships through a probability matrix ***π***, where *π*_*kl*_ affects the edge connection probability between two nodes in communities *k* and *l*. Assuming no corrections due to node degrees (degree-corrected versions also exist^15^), one seeks a partition, **z**, which maximizes,

4 $$p({\bf{A}}|{\bf{z}},{\boldsymbol{\pi }})=\prod _{i\ne j}^{N}\,\prod _{k,l}^{K}\,{({\pi }_{kl}^{{a}_{ij}}{(1-{\pi }_{kl})}^{1-{a}_{ij}})}^{\delta ({z}_{i}=k,{z}_{j}=l)},$$

where *δ*(*z*_*i*_ = *k*, *z*_*j*_ = *l*) is 1 if *z*_*i*_ = *k* and *z*_*j*_ = *l*, and 0 otherwise. One can maximize this objective with the expectation maximization (EM) algorithm, belief propagation, and efficiently with an iterative approach which, similar to Louvain, agglomerates blocks of nodes at each iteration^6^. Specifically, at each iteration a Metropolis-Hastings accept-reject sampling approach is used to compute a probability of merging blocks based on how the merge affects the likelihood.

## Limitations of Community Detection

These agglomerative heuristics for both modularity and likelihood maximization simplify a computationally challenging task but can still be time consuming for large networks and often give rise to large variability in the partitions returned across multiple runs of the algorithms. We seek to explore how a compressed network representation can improve these issues.

Motivated by super pixels, we wish to define seed nodes in networks that can be used as a starting point to grow out ‘super nodes’ to define a new, smaller network upon which we apply standard community detection algorithms. Creating a direct analog of super pixels in networks is challenging because the inherent geometry of a network can be quite different from the grid layout of an image (where simple neighborhood structures such as 4- or 8-neighborhoods are typically used), and we need to ensure seeds are well distributed across the network. Further, while super pixels are largely constrained by the structure of the pixel grid (i.e. proximity between pixel pairs matter), their definition also incorporates extra image features to refine members of a super pixel set, whereas in network community detection we typically only have the edges of the network to work with. Finally, the performance of a super pixel representation of an image can be objectively validated from the quality of the corresponding segmentation result, with reference to human-specified objects in images; in contrast, community detection is typically an unsupervised, exploratory data analysis technique with limited available notions of ‘ground truth’^16–18^. As such, we must develop measures that can be used to validate the quality of the super node representation.

## Related Work

Our objective to define a smaller network of super nodes is a form of network compression. Several references have explored useful ways to compress networks^19–24^, with Yang *et al*.^20^ and Peng *et al*.^22^ using graph compression in the context of community detection. A review of network compression and summarization techniques is given in ref.^25^. These compression approaches can either be classified as *network pre*-*processing* or *network size reduction*. Under these definitions, pre-processing refers to a method that uses all of the nodes to pre-partition the network or agglomerate nodes to form a smaller network of pre-agglomerated nodes or’super nodes’. Creating a super node representation of the network can assist in visualization, gives control over how many nodes to split the network into, and allows for the input of a pre-processed network into standard network analysis tools. Alternatively, in network size reduction approaches, nodes are systematically removed and further analysis is performed on a smaller subnetwork. Such an approach may be useful if one has prior knowledge of unimportant or redundant nodes. Two network pre-processing methods that define super nodes are explored by Yang *et al*.^20^ and Lisewski *et al*.^19^; but these approaches differ from our proposal in that they seek to define super nodes along with additional side information about relationships between node pairs. First, Lisewski *et al*.^19^, describes ‘super genomic network compression’ to reduce the number of edges in a large protein interaction network. To do this, the authors identify ‘clusters of orthologous groups’ of proteins, or proteins that give rise to similar functions in different species and originated from a common ancestor. Members of an orthologous group are connected as a star network, with the center node as one member of the orthologous group. Furthermore, edges between orthologous groups are replaced by a single weighted link reflecting the pairwise group evolutionary similarity. Next, Yang *et al*.^20^ define super nodes by specifying ‘must link’ and ‘cannot link’ constraints between pairs of nodes, agglomerating as many nodes as possible sharing must link constraints while being cautious about agglomerating nodes that cannot link. Finally, Slashburn introduced by Lim *et al*.^23^ is another pre-processing approach for network compression that seeks to identify a permutation or ordering of the nodes, such that the adjacency matrix is pre-processed to have sets of clustered edges. To accomplish this task, hubs are removed iteratively and nodes are re-ordered so that high degree nodes appear first in the ultimate ordering of nodes.

Alternatively, approaches that perform network compression through network size reduction were presented in three works^21,22,24^. Gilbert *et al*., introduce the ‘KeepAll’ method^21^, which seeks to prioritize a set of nodes according to their importance in the network and retain only the smallest set of additional nodes required for the induced subgraph of prioritized nodes to be connected. Results in this paper highlight the method’s ability to remove redundant and noisy nodes that allow for clearer analysis of the original set of prioritized nodes. Peng *et al*.^22^ extract a smaller network through a *k*-core decomposition, and perform community detection on the subnetwork. While we also seek to perform community detection on a smaller version of the network, we seek to do this in the network pre-processing manner so that all nodes are effectively included as the input to the community detection algorithm, with flexibility to choose the number of super nodes or size to represent the network with. Given that the number of nodes in the *k*-core of a network decreases dramatically with an increasing *k*, there is not much flexibility in the scale or size of the network representation. Finally, Liu *et al*. also use a *k*-core based approach to decompose the network in a different manner. The authors define CONDENSE^24^, an information theoretic based method to reduce a large network into a set of representative substructures. In particular, induced subgraphs resulting from the *k*-core based clustering technique are each treated as representative substructures.

## Defining Super Nodes

**Figure 1:**
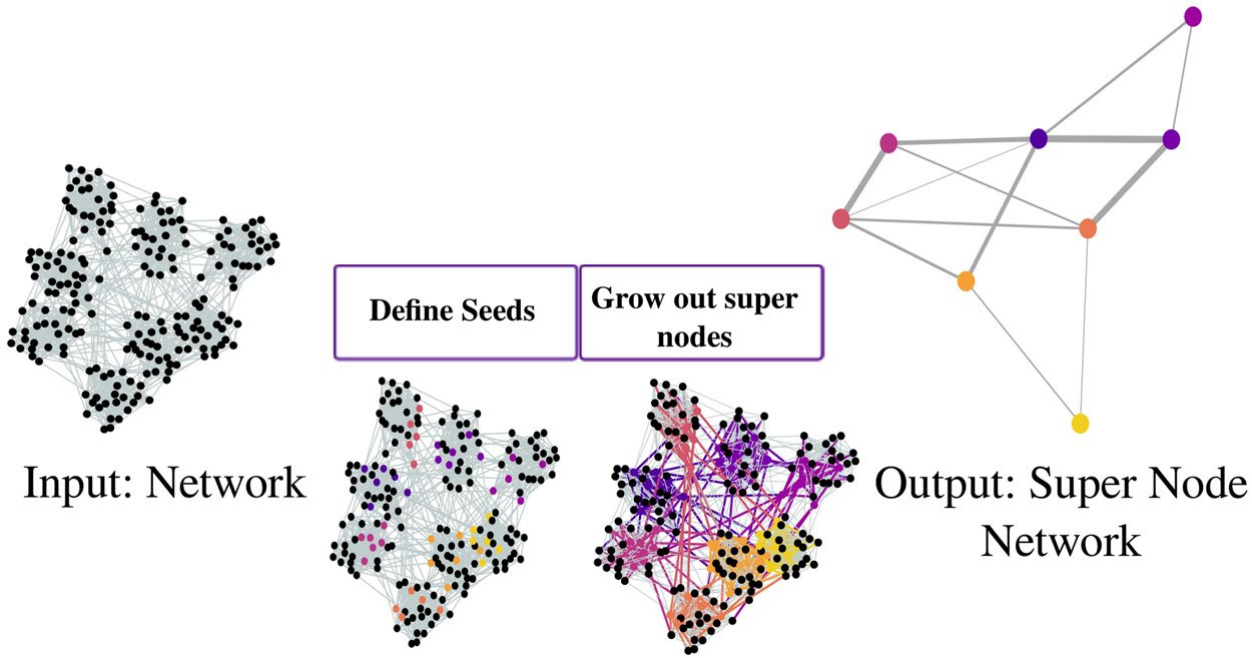
Defining super nodes. To define the super node representation of a network, we select *S* seeds and agglomerate local regions around them to create super nodes. This then leads to a new network with weighted edges between the *S* super nodes upon which community detection can be more efficiently applied.

To define the super node representation of an unweighted *N*-node network, we first select $S\ll N$ seed nodes through a 2-core decomposition (discussed further in Methods). We then agglomerate the remaining *N* − *S* nodes around the seeds to create super nodes. Finally, we specify the network between these super nodes. Community detection can then be applied to the *S*-node network representation. Figure 1 visualizes this approach, with details provided in the Methods.

The aims of this work are twofold: *First*, we seek to develop an effective way to define a super node representation of a network that can then be used in standard community detection algorithms, such that the representation minimizes our defined under segmentation error and maximizes NMI with the partition that would have been obtained using the full network. *Second*, we wish to highlight several benefits of using such a compressed representation of the network in community detection tasks. In particular, we show that a super node representation of the network accomplishes the following.

1. **Decreased runtime for community detection**: Even though recently developed heuristics for maximizing modularity^7^ and fitting SBMs^6^ are highly efficient relative to previous approaches for performing the same computational optimizations, these methods can still be time consuming for large networks. We aim to reduce runtime for large networks, moving most of the computational cost in practice from tasks scaling with the size of the network to alternatives scaling with the (much smaller) size of the super node representation.
2. **Decreased stochastic variability of community detection algorithm output**: In large networks, there is often significant variability across multiple runs of the same algorithm (employing computational heuristics to solve NP-Complete optimizations), as well as differences between various community detection algorithms. We expect applying community detection to a well-chosen super node representation to decrease the observed variability.
3. **High local agreement**: In defining super nodes, we inherently assume that the identified communities should agree with the local network connectivity in that members of a neighborhood should be more likely to have the same community assignment, provided that the super nodes were constructed to minimize aggregation across community boundaries.
4. **Consistent with communities found using the full network**: Despite the differences in line with the above features, the identified community structure should still be relatively similar to the distributions of results that would have been obtained through applying community detection to the full network.

## Results

To demonstrate the effectiveness of super nodes, we performed several experiments to analyze the runtime, partition variability, alignment of communities with local network connectivity, and alignment of super node representation and full network communities. We considered 9 unweighted network data sets (see Table 1) from the Stanford Network Analysis Project database^26^ (Enron, Amazon, Dblp, Email, BrightKite, Stanford, Notre Dame) and Newman’s collection^27^ (As22, CMatter). We treat all networks as undirected. Note that some of these networks are large subgraphs extracted from the original networks. In particular, these subgraphs are defined by the union of all nodes of degree ≥2, their neighbors, and next nearest neighbors. We use the Louvain algorithm (https://github.com/vtraag/louvain-igraph) for modularity maximization^7^ and the stochastic block model (SBM) inference (https://graph-tool.skewed.de) described in ref.^6^. Since the super node representation ultimately produces a weighted network, where the edge weights are counts computed based on the original network, both of these community detection algorithms are able to accommodate these kinds of edge weights.

**Table 1:** Network data characteristics.

Dataset (*Indicates subgraphs)	#Nodes	#Edges
CMatter* (Condensed matter 2003 collab.)	17,816	83,337
As22* (Internet)	22,801	48,270
Enron*	32,374	178,195
BrightKite (loc-BrightKite)	58,228	214,078
Amazon* (com-Amazon)	77,463	209,887
Dblp* (com-DBLP)	150,801	639,330
Email (email-EuAll)	265,214	420,045
Stanford (web-Stanford)	281,903	2,312,497
Notre Dame (web-Notre Dame)	325,729	1,497,134

**Figure 2:**
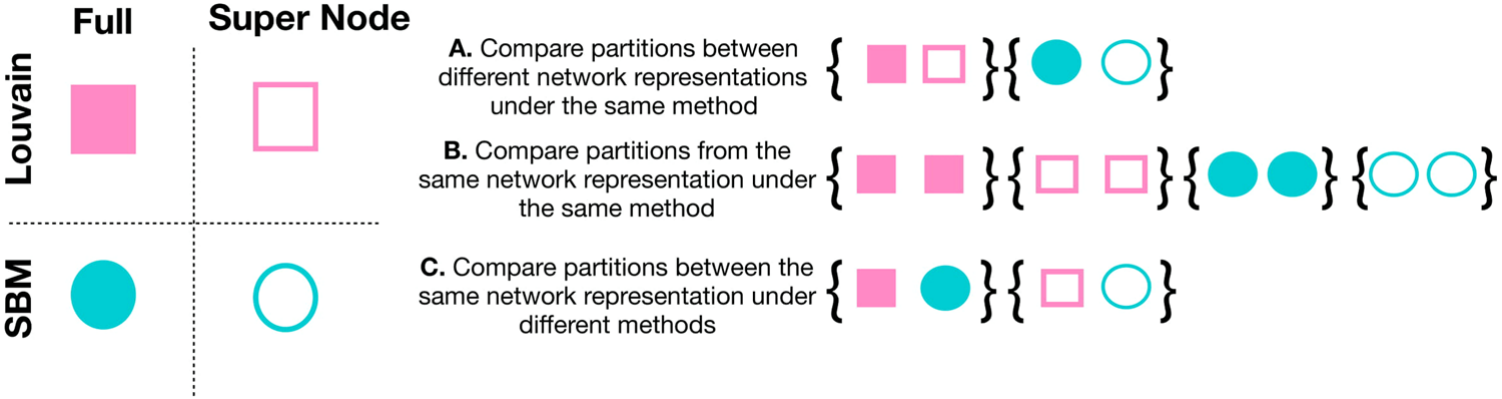
Schematic of possible partition comparisons. We outline the types of possible comparisons between partitions generated according to various combinations of network representation and community detection method. According to a particular comparison rule, we compute normalized mutual information (NMI) between all pairs of networks satisfying the comparison description. The unique symbols in the schematic correspond to a community detection partition of the network obtained according to a particular algorithm and network representation combination. Partitions obtained with the Louvain algorithm are denoted by pink squares, while partitions with an SBM are shown as blue circles. Network representation is coded by solid symbols for the full network and hollow symbols for the super node network. In (**A**–**C**) we outline the types of comparisons we perform in subsequent figures. (**A**) To compare the usefulness of the super node representation in identifying communities retrieved using the full network, we compare pairs of networks with different representations under the same community detection algorithm. (**B**) Due to the stochastic nature of both the Louvain algorithm and SBM fitting, this comparison seeks to quantify partitions generated under the same network representation and method. (**C**) Finally, we consider pairs of partitions generated under the same network representation and different community detection algorithms.

Because we consider a variety of comparisons between partitions under multiple community detection methods and network representations, we provide a schematic in Fig. 2 of the performed comparisons in subsequent figures. In these comparisons, we use normalized mutual information (NMI) to quantify the similarity between a pair of partitions. In general, there are four possible combinations of community detection method/network representation that can be applied to identify communities. First, there are two choices of community detection algorithms, Louvain algorithm or stochastic block model fitting. There are also two choices for network representation, which is to use either the full network or super node network representation. In Fig. 2, we first visualize the community detection algorithm and network representation combinations with different types of symbols and colors. Each symbol is intended to represent a community detection produced partition of the network under a particular method/network representation. For example, pink squares are partitions obtained under the Louvain algorithm. Blue circles symbolize partitions according to the stochastic block model. The network representations used, full network or super node representation, are denoted by filled and hollow shapes, respectively. We assume that under each network/method combination, we can generate multiple partitions that will be somewhat different from each other. Amongst all partitions, we perform our analyses on all pairs of network partitions that satisfy the network/method combination specified by each of the eight comparisons represented in Fig. 2. Comparisons between pairs of networks (shown in brackets in the Figure) fall into one of three types. The first partition comparison we consider is shown in Fig. 2A, and seeks to quantify the similarity between a set of partitions generated with different network representations under the same community detection algorithm. The comparison computes NMI(**z**^*Full*^, **z**^*SN*^). For example, we consider the pairwise similarity between the full network and super node representations with the stochastic block model. This comparison helps to understand how well the super node representation yields the node-to-community assignments obtained using the full network. Next, we explore the inherent variability of community detection algorithms, which seems to especially arise among partitions of a large network. Figure 2B considers pairs of partitions under the same network representation and community detection algorithm. For example, we run the Louvain algorithm on the super node representation of the network multiple times and compare all pairs of partitions. Finally, in Fig. 2C, we consider pairs of partitions generated under the same network representation but with different methods. In one example, we compare partitions of the full network representation, where one partition used the Louvain algorithm and the other is obtained by fitting an SBM. Even though these methods are different by design, we want to measure the extent to which they capture common structures.

### Objectively Comparing Partitions on Possibly Different Scales

A challenge in directly comparing the community partitions on the full and super node network representations is the difference in scales between the partitions. For example, using the full network typically produces significantly more communities than under the super node representation. In an attempt to compare community partitions with similar size distributions in the subsequent experiments, we can choose algorithm parameters to adapt the scales of the partitions obtained from the Louvain algorithm and SBM fitting. Conceptually, this is achieved by using the super node representation of the network to choose a comparable parameter to apply during the community detection task on the full network.

In community detection with the Louvain algorithm, we identified comparable resolution parameters (controlling community size) to apply to the full network that produce a size distribution agreeing as much as possible with the community partition in the super node network. We compute experimental results using both the default resolution parameter and the ‘matched’ parameter. While the default resolution parameter is *γ* = 1, in our analyses we computed partitions of the full network using several different $\gamma \in [0.05,\,2.5]$. To choose the matched resolution parameter on the full network, we first find the community partition using the super node representation. For each partition, we then order nodes based on the sizes of the communities to which they belong. With this approach, all nodes from the same community are at the same position in the ordering. For each partition of the full network (at different resolution parameters), we then consider the similarity of this ranking with that from the super node communities, measuring this similarity by Kendall’s tau correlation. We identify the resolution parameter producing the highest Kendall’s tau correlation, referring to this resolution parameter as the ‘matched parameter’ in the remainder of the text, while we refer to the standard *γ* = 1 as the ‘default’ resolution parameter.

In fitting SBMs, we chose to fit a model with the same number of blocks that was found in the super node representation using the standard optimization and model selection strategies discussed in ref.^6^. We refer to the ‘matched’ version as that using the number of blocks identified by the model selection on the super node representation, while the ‘default’ result is obtained using the model selection strategy on the full network. In subsequent experiments, we compare both the ‘matched’ and ‘default’ versions to ensure our results are not artificially influenced by the scale of the community sizes.

### Experiments

**Figure 3:**
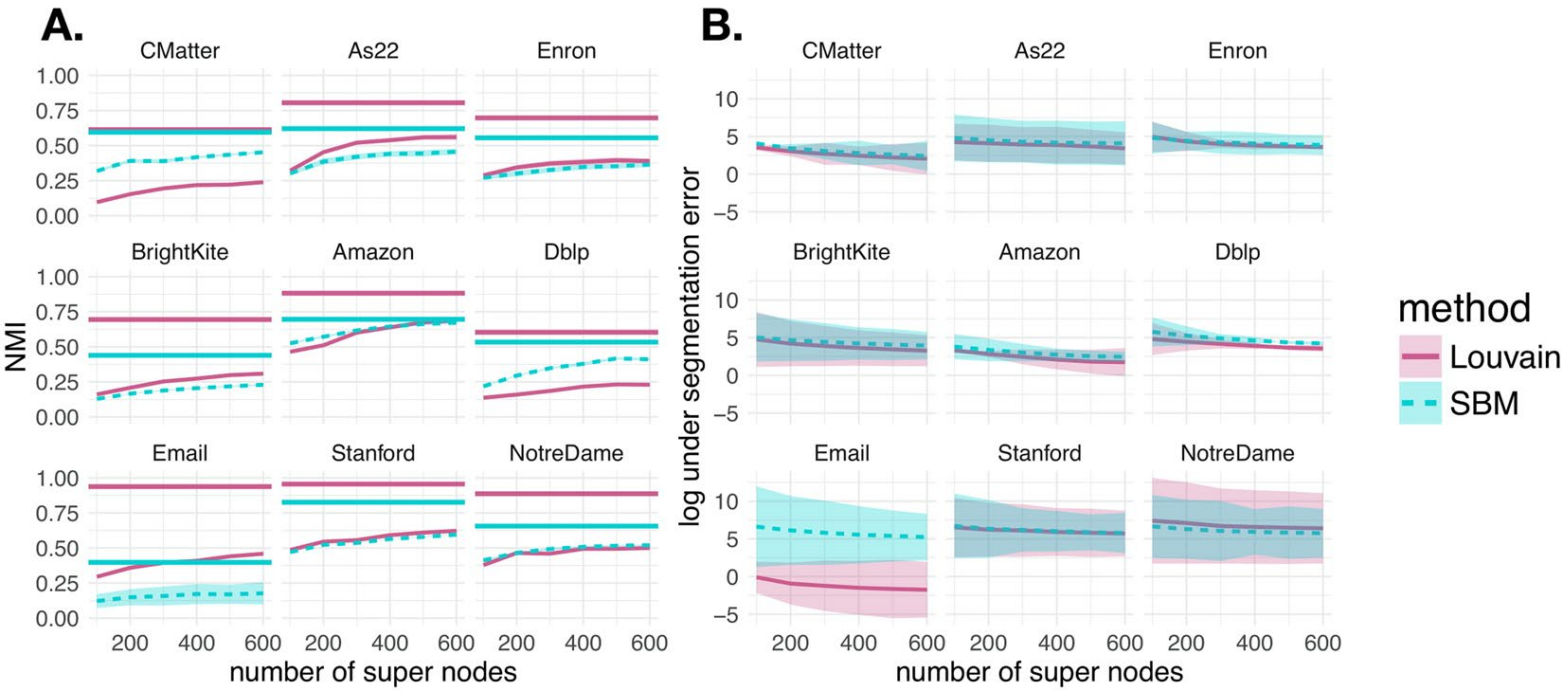
Super Node Quality. We computed normalized mutual information (**A**) and under segmentation error (**B**) for networks represented by between 100 and 600 super nodes. Line type and color indicate the community detection algorithm applied (Louvain algorithm or SBM fitting). Each curve indicates the mean across 5 super node representations. The shaded area shows standard deviation. (**A**) Normalized mutual information between the full and super node representations of networks [i.e. NMI(**z**^*Full*^, **z**^*SN*^)]. A network representation with more super nodes. generally increases the NMI between full network and super node network representations. Horizontal lines indicate the mean pairwise NMI between 10 runs of the Louvain algorithm and SBM result on the full network (pink and blue, respectively). Given the high variability between multiple runs of the same algorithm on the full network, adding more super nodes can only improve the NMI between the full and super node representation. (**B**) The log under segmentation error for super node representations. Defining a super node representation with more super nodes generally decreases the under segmentation error.

First we measure the quality of the super node representation in terms of NMI and under segmentation error that were defined in equations 1 and 2. In Fig. 3, we vary the number of super nodes and examine the normalized mutual information (A.) (equation 2) and log under segmentation error (B.) (equation 1) in each of the 9 networks. The curves represent the mean NMI (A.) and mean under segmentation error (B.) over 5 super node representations, with the shaded area denoting standard deviation. Varying the number of super nodes between 100 and 600, the results generally indicate that as the number of super nodes increase, the network has 1) lower under segmentation error and 2) higher NMI (i.e. similarity) with the partition obtained using the full network. Each curve and line type in Fig. 3 specifies whether community detection was performed using the Louvain algorithm or by fitting an SBM. Note that this comparison between the partition with the full network and the super node representation within each method corresponds to Fig. 2A. To give some intuition about what value of NMI is considered good, we put our results in the context of the partition variability among 10 runs of the same community detection algorithm. In Fig. 3A, we indicate the mean pairwise NMI between multiple runs of the Louvain algorithm and SBM fitting with horizontal pink and blue lines, respectively (as described in Fig. 2B). For the most part, the pairwise NMI between partitions is not 1. Therefore, by increasing the number of super nodes, we can only expect to asymptotically approach the mean pairwise NMIs between multiple runs of the same algorithm on the full network. Randomly permuting the node-to-community assignment obtained under the super node representation (**z**^*SN*,*perm*^) 1000 times and computing the NMI with the full network (i.e. NMI(**z**^*Full*^, **z**^*SN*,*perm*^)) gives a mean NMI of approximately 0.01. Another interesting observation is that for both the Louvain algorithm and the SBM, the NMI results level out at between 300 to 400 super nodes.

**Figure 4:**
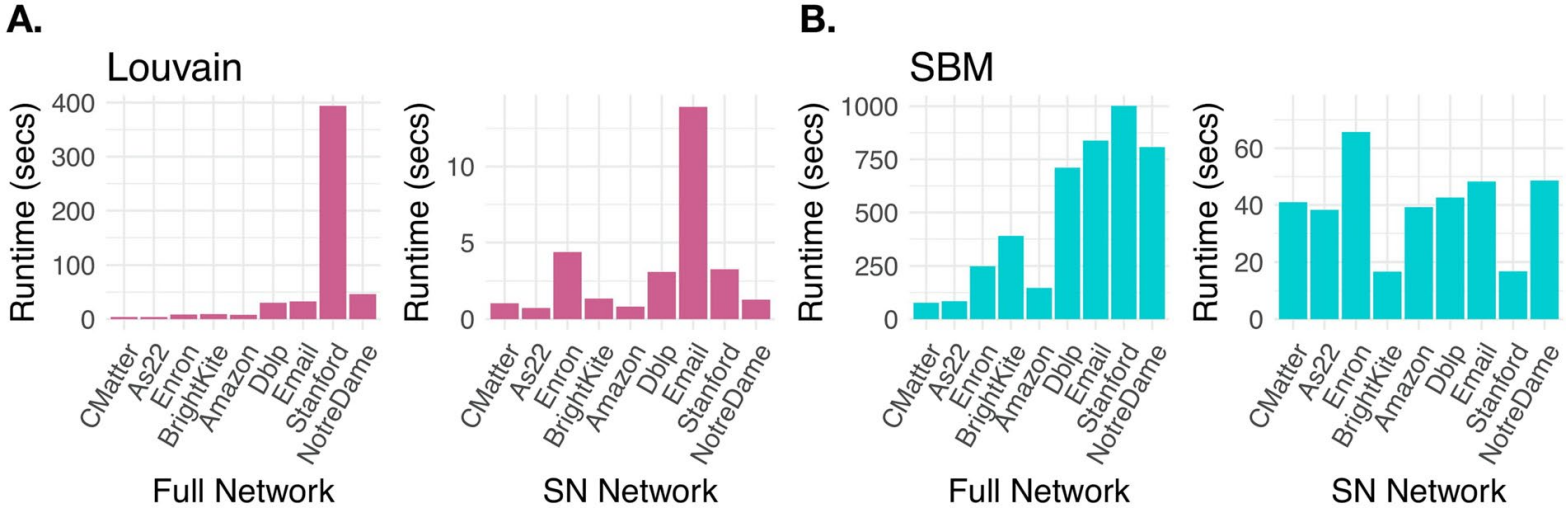
Runtimes. We compare community detection runtimes (in seconds) with the Louvain algorithm and by fitting an SBM on the full networks and super node representations for the 9 data sets. (**A**) Louvain on the full network. (**B**) Louvain on the super nodes. (**C**) SBM on the full network. (**D**) SBM on the super nodes.

In practice, one of the most desirable properties of a super node representation of the network is the decrease in the run-time of community detection algorithms in comparison to using the full network. In Fig. 4, we recorded the runtime required to identify communities with the Louvain algorithm and stochastic block model inference procedure under the full a 500 node super node network representations in each of the 9 networks. The Louvain algorithm is fast and scales well, at *O*(*M*) per iteration for *M* edges, with its relative speed and high modularity values contributing to its popularity. While the reported runtimes may seem quite modest, in practice it is common to run many realizations of the algorithm (hundreds, thousands, or even more for large networks) to explore resolution parameters and stochastic variation due to pseudorandom node order in the heuristic. We note a large increase in runtime for the full Stanford network, with over 2 million edges. As also observed in the figure, fitting a stochastic block model, at $O(N\,{\mathrm{ln}}^{2}\,N)$ for sparse networks in this implementation^6^, becomes significantly slower on the full networks with more than 200,000 edges.

While we see a significant improvement in community detection runtime from using super nodes for both methods, the benefit in the SBM fitting is particularly large, especially for the bigger networks (Dblp, Stanford, Email). In moving to the super node representation, we traded out large-coefficient scaling-with-*N* community detection computations for those scaling with $S\ll N$ (with possible increases due to the increased density of the super node representation), at the cost of constructing the super node representation. In particular, we observe the SBM runtimes on super nodes appear to be relatively independent of *M*. We note that each of the three steps building our super-node representation is *O*(*M*), so in the large graph limit the expected gain of our approach may be only a constant factor over Louvain iterations and the SBM fitting (up to logarithmic factors). In the present calculations, we have not endeavored to optimize the runtime to build our super node representations; even so, the three steps building the *S* = 500 super node representation of the Stanford network in our current implementation together take ~350 sec with CoreHD and ~200 sec using highest-degree nodes. While this alone might not seem like a large improvement compared to a single realization of running Louvain or fitting an SBM, the computational gains compared to generating multiple community partitions can be quite significant.

**Figure 5:**
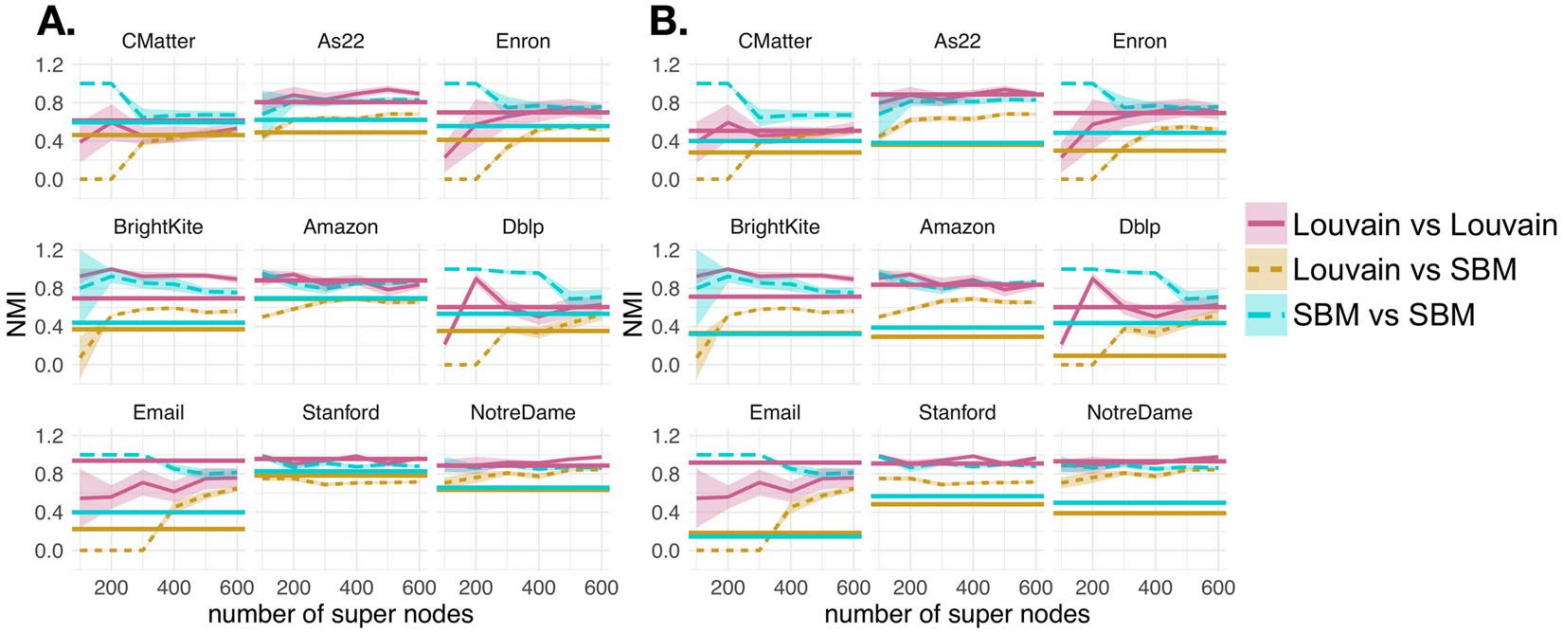
Quantifying partition variability. For each of the 9 networks, we obtained 10 different partitions by the Louvain algorithm and 10 different SBM fits under the default (**A**) and matched settings (**B**). To assess the similarity between partitions within and between community detection algorithm in the constructed super node networks, we computed pairwise normalized mutual information (NMI) as a function of the number of super nodes. The pink and blue curves show the mean pairwise normalized mutual information between all pairs of 10 partitions under Louvain and SBM fitting, respectively. The gold curves compare pairs of partitions under different methods. Shaded area denotes standard deviation. Horizontal lines indicates the mean pairwise NMI between partitions under the full network representation for within Louvain and SBM partition comparison (pink and blue, respectively) and between Louvain and SBM partition comparison (gold). Overall, the super node representation is useful for reducing the disparity between the partitions obtained under different methods.

Next, we present evidence that there is variability in the partitions generated by multiple runs of the same community detection algorithm on the same network representation. We sought to quantify how the variability or pairwise similarity between multiple runs of the same algorithm under the super node representation changes as a function of the number of super nodes. Furthermore, we directly compared these results to variability observed in the full network under the default and matched algorithm parameters (Fig. 5A,B), respectively. Curves represent the mean pairwise NMI between all pairs of 10 computed partitions under the super node network representation and shading shows standard deviation. The pink and blue curves show the within-method comparisons on the super node network representation for the Louvain algorithm (‘Louvain vs Louvain’) and stochastic block model (‘SBM vs SBM’), respectively (comparison referenced in Fig. 2B). We were also interested in the variability of the partitions obtained between partitions of the super node representation found with different algorithms. This result is shown in the gold curve and labeled ‘Louvain vs SBM’ (comparison described in Fig. 2C). The horizontal lines show the mean pairwise similarity observed between all 10 runs of the Louvain algorithm and stochastic block model (pink and blue, respectively) on the full network (comparison Fig. 2B). Similarly, the horizontal gold line shows the mean pairwise similarity between all runs of the Louvain algorithm and SBM fitting on the full network (comparison Fig. 2C). Note that the curves are the same in both A. and B. because the matched and default parameters are incorporated only on the full network to best match the scale of the super node representation. The most significant improvement we observe under the super node representation is between runs of the Louvain algorithm and SBM fitting, suggesting that the new compressed representation of the network has prominent structural features that are robustly identified with both approaches. A high normalized mutual information between a pair of partitions indicates that the algorithms identified similar community structures. The Louvain algorithm is generally less variable than fitting an SBM, but we also observed decreased variability in the fitting of stochastic block models on the super node representation.

**Figure 6:**
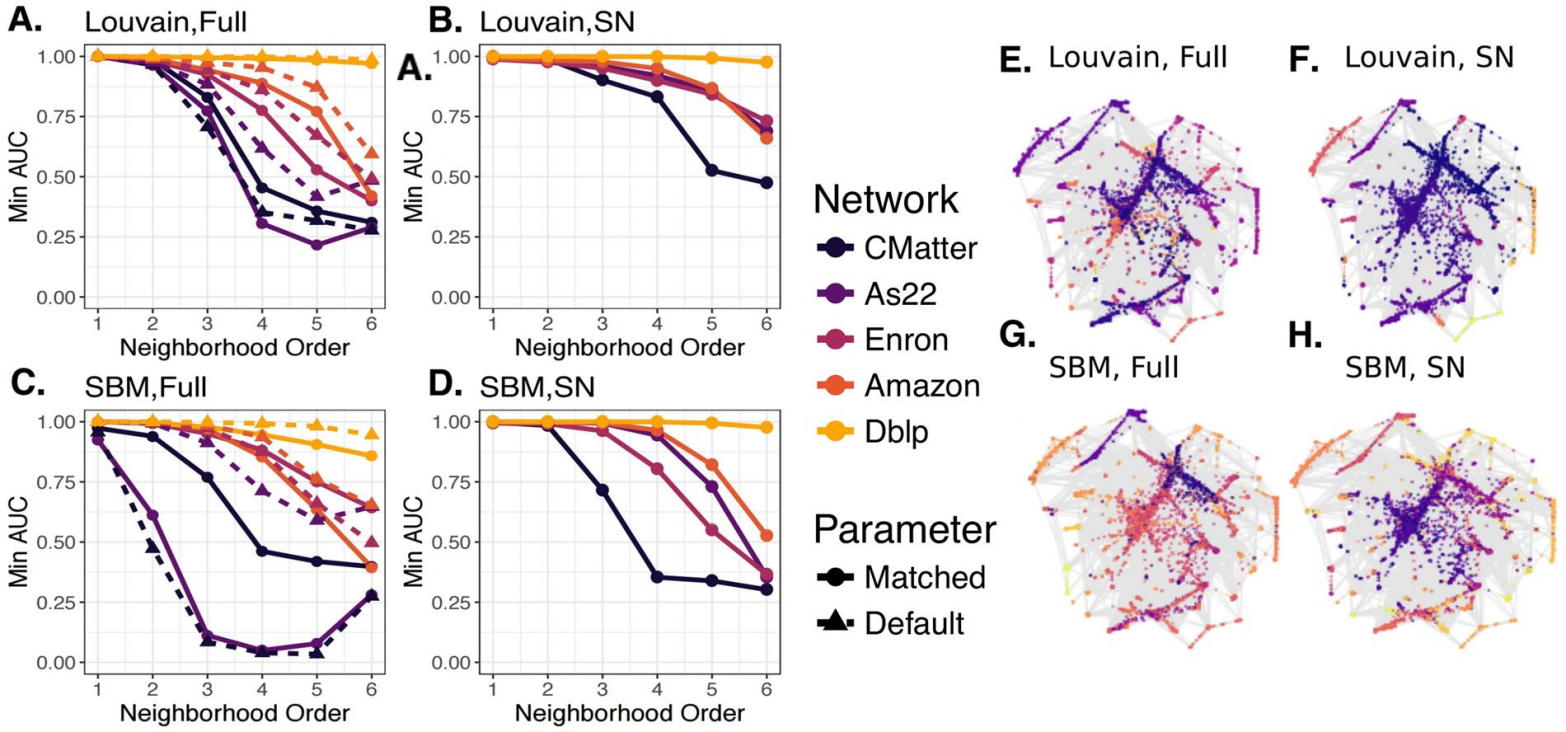
Agreement of community assignments with local connectivity. We study how consistent partitions are within local neighborhood regions of the network by examining how well a node’s neighbors (for various order neighborhoods) can be used to predict its community assignment, under some community partition **z**. For each community in a partition, we give a binary prediction of whether a node is assigned to that community, based on probabilities we compute for a node from its neighbors. Sweeping the parameter *p* that sets the probability required for a node to be assigned to a community, we compute ROC curves for each community and report the minimum AUC value observed. Panels (A–D) show minimum AUC values observed as a function of neighborhood order for communities obtained from the full networks and super node representations by Louvain and by SBM. Line color indicates network and line type indicates communities obtained from the matched and default parameters used by the algorithms on the full networks. Panels (E–H) visualize the communities obtained in the As22 data on the full network (default parameters) and super node representation (SN) under Louvain and SBM, with node colors indicating community memberships.

While we have emphasized benefits in the mechanics and usability of running standard community detection algorithms, we now seek to address whether the communities that we find using the super node representation align with local network connectivity so that neighbors are more likely to have similar community assignments and how this alignment compares with what we would have found by community detection on the full network. While we visualize this qualitatively for the As22 network in Fig. 6E–H, we also designed a prediction task to quantify this alignment. In this prediction task, we seek to take a node-to-community partition (from either the full or super node network representations **z**^*Full*^ or **z**^*SN*^, respectively) and the full network ${\mathscr{X}}$ to see how accurately we can predict members of a community for different neighborhood sizes. For network ${\mathscr{X}}$ with node-to-community assignments **z**, we assign a probability distribution to each node over all of the communities under **z**. For a neighborhood order *o* (x-axis in Fig. 6), we say that node *i* has probability of being in community *k*, based on what fraction of its neighbors belong to that community under **z**. Then for each community in **z**, we perform a binary prediction task for whether each node of ${\mathscr{X}}$ should be assigned to that community, according to the computed probability distributions for all nodes with respect to that community.

We sweep the probability parameter, *p*, representing the required threshold probability for a node to achieve in order to be assigned to a community in this binary classification task. By sweeping *p* for each of the communities, we compute an ROC curve for each community and the corresponding areas under the curve (AUC). Finally, we use the minimum AUC value as our summary statistic of this task, with a high AUC value indicating that the neighboring regions of a node were strong predictors of community assignments, as shown in Fig. 6A–D. All experiments are performed on 5 networks (As22, Enron, CMatter, Dblp, Amazon) and for both the matched and default parameters (indicated by line type) in the full network. (Recall from section 3.1 that the matched parameters for the full network were chosen based on the super node partition results under default settings; hence, there is no corresponding ‘matched’ set for the super nodes in these plots.) We observe in most cases using the super node representation improves the minimum AUC value, indicating that communities obtained from this representation have higher agreement with local connectivity by this measure.

To qualitatively evaluate how the super node representation is able to ultimately partition the network into communities that are locally relevant, we visualize the As22 network, with nodes colored by communities identified under the full network (E,G) and super node representation (F,H) under Louvain (E,F) and SBM (G,H). Consistent with the quantitative results in Fig. 6A–D, we observe that the super node representation leads to an effective coarse-graining, with the community labels appearing to be qualitatively consistent across large regions of the network.

### Comparison with other network compression methods

Thus far, we have shown how our approach to defining super nodes can be used to identify communities with high agreement to the result obtained using the full network. As we described in the beginning of this paper, the desire to compress networks is an area of active research, so we sought to compare the performance of our approach to some of the state-of-the-art methods for this task. We focused on comparison with two alternative methods, due to their publicly available code and for being straightforward to implement. First, we compare our results to seeds selected with Slashburn^23^, a node reordering method suited for network compression. Second, we compare to *k*-core based clustering (KCBC), introduced in ref.^24^. Here, we will briefly define each method and how we used it for the task of defining super nodes.

#### Slashburn

Slashburn^23^ is a node reordering algorithm used for graph compression. This method uses hubs to decompose a network into connected components. Formally, Slashburn seeks to identify the ordering of the nodes such that a specified storage cost function is minimized. Iteratively, high centrality nodes are removed, while high degree nodes are ranked earlier in the ordering and low degree nodes are ranked later. We used the implementation of Slashburn available on github (https://github.com/theeluwin/bear). The input to Slashburn is the original network, **A**, and the output is a permutation of the node indices. From the Slashburn ordering of nodes, we defined seeds around which to grow our *S* super nodes by considering the top *S* Slashburn ranked nodes. Related to our approach using CoreHD, we used Slashburn in this context as a method to select seeds. For the problem of identifying representative network structures described in ref.^24^, the authors used Slashburn to identify meaningful network substructures. In particular, they found substructures by considering the ego networks of the highly Slashburn ranked nodes.

#### KCBC

The *k*-core based clustering introduced by Liu *et al*.^24^ is an important part in their proposed network summarization method, ‘CONDENSE’ (Conditional Diversified Network Summarization). The overarching objective of CONDENSE is to concisely describe the network with a set of subgraph structures in such a way that achieves minimum description length. To identify the set of substructures, the authors decompose their network through a *k*-core decomposition. To do this, *k*-cores are iteratively removed, starting by setting *k* equal to the max core number. At each of these steps, each connected component resulting in the present *k* is denoted as a *decomposition set* and treated as an induced subgraph. Edges in the network between nodes in the decomposition set are then removed. This process is repeated until all edges in the original network have been removed. Furthermore, each of the connected components extracted in this process comprises one member the set of representative network structures. We use KCBC to define seeds for our super node centers by extracting the highest degree node (according to degree in the full network) in each of the identified substructures. Note that unlike our own seed selection method and the Slashburn seed selection approach, we do not control how many super nodes to use to represent the network. Instead, we use only as many super nodes as there are substructures under the *k*-core based clustering approach.

**Figure 7:**
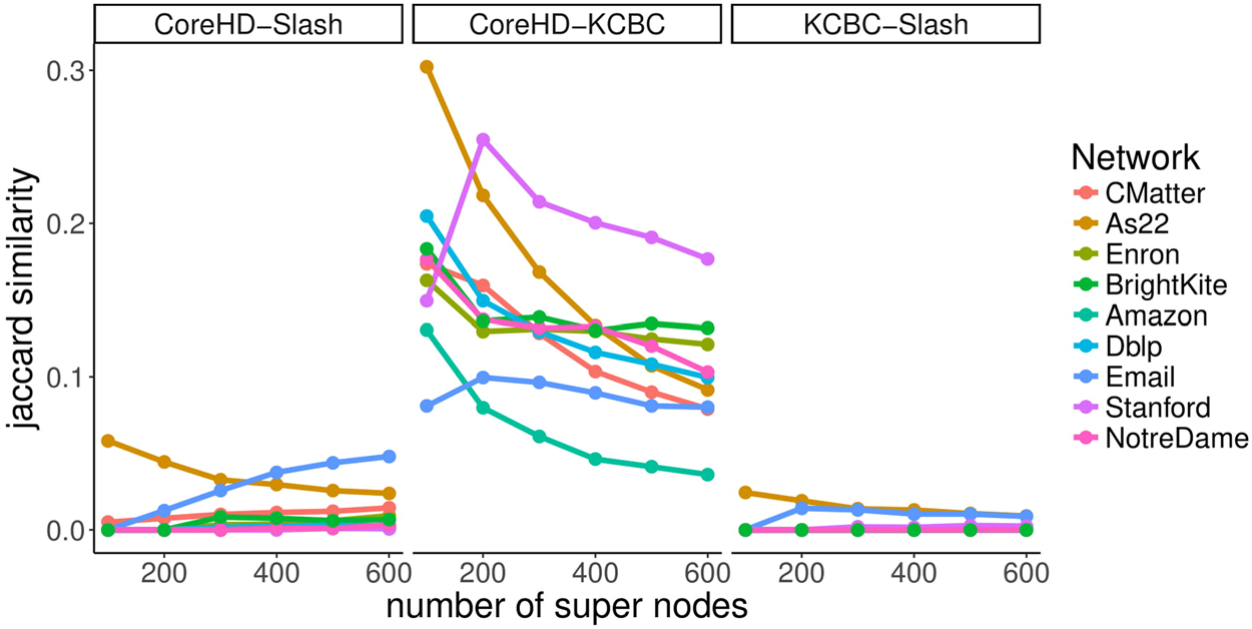
Comparing methods for finding seeds. We identified seeds in each of our 9 networks using our Core HD based approach (CoreHD), Slashburn (slash), and *k*-core based clustering (KCBC). Jaccard similarity was used to quantify the similarity between each set of seeds (S*) returned by each method for varying numbers of super nodes. We observed the strongest similarity between our 2-core based seed selection approach and KCBC.

First, we simply compared the set of seeds selected under CoreHD to the seeds obtained with Slashburn (denoted Slash) and through *k*-core based clustering (KCBC). For each of our 9 networks, we computed a set of seeds under each of the three methods and then computed the Jaccard similarity between the set of super nodes (S*) under each possible pair of the three methods. We considered sets of seeds from *S* = 100 to *S* = 600. As described above, we did not explicitly specify the number of super nodes, *S*, to identify with KCBC. These results are shown in Fig. 7. Each column represents one of the three possible pairwise comparison between the three methods. We noticed the strongest similarity between our method of finding seeds and KCBC. In general though, the sets of seeds returned are quite distinct.

**Figure 8:**
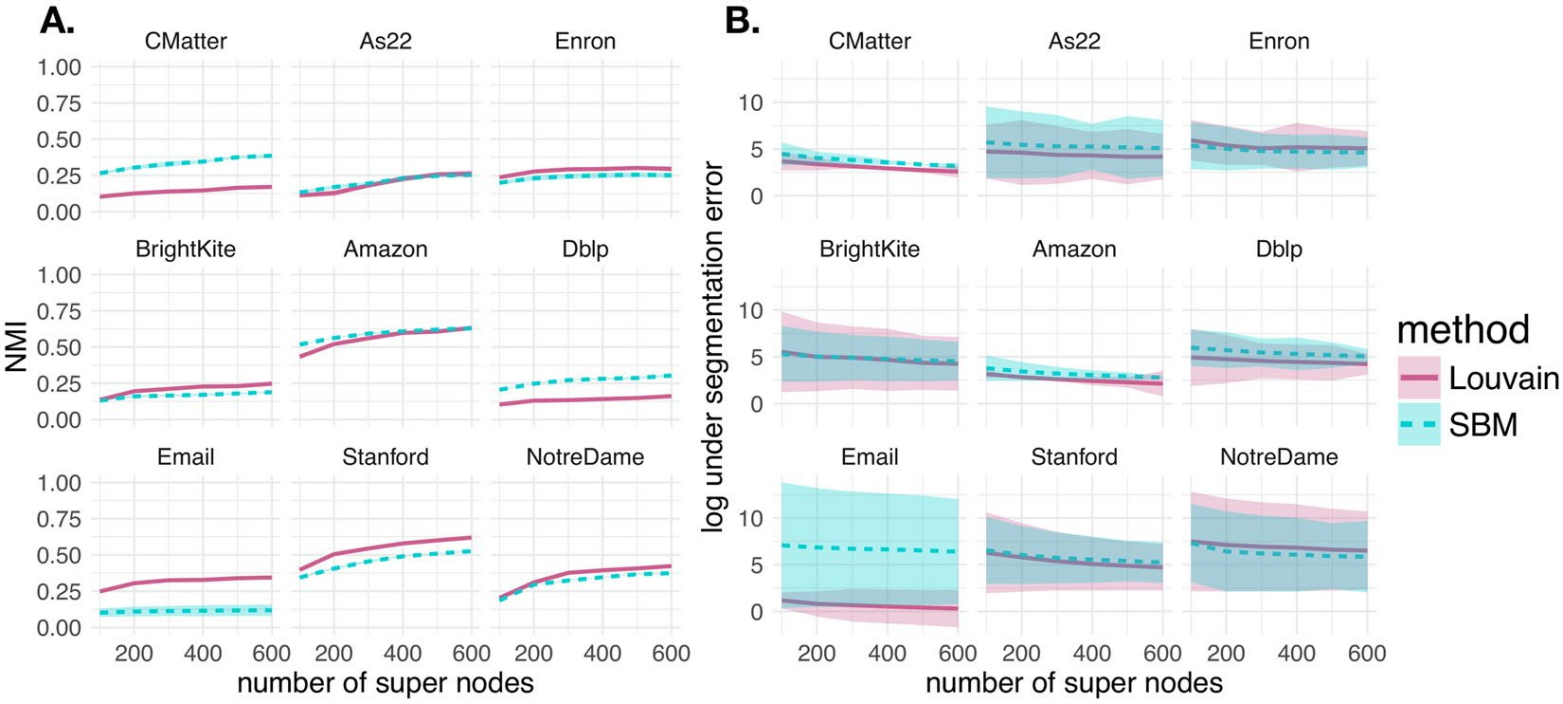
Super node quality with slashburn. Seeds were defined using Slashburn to result in network representations of the original network between *S* = 100 and *S* = 600 super nodes. We performed the super node quality analyses shown in Fig. 3, profiling (NMI(**z**^*Full*^, **z**^*SN*^)) (**A**) and under segmentation error (**B**). Each curve represented the mean value across 5 runs of each experiment and shaded area denotes standard deviation. When considering the maximum mean NMI value observed across various numbers of super nodes, the CoreHD approach and often the Slashburn approach tend to produce higher NMI values. Under segmentation errors are similar across each of the three approaches.

Next, we sought to perform the analysis of super node quality described in Fig. 3 for the Slashburn and KCBC methods. Similar to the results presented in Fig. 3, we also performed analysis on NMI(**z**^*Full*^, **z**^*SN*^) and under segmentation error among super node results defined under CoreHD and Slashburn. Overall, we find the results on partition agreement (NMI(**z**^*Full*^, **z**^*SN*^)) to be qualitatively similar regardless of how seeds are chosen. However, we note that for each of the 9 networks seeds chosen with the Core HD method resulted in higher agreement between community detection results on the full and super node network representations (NMI(**z**^*Full*^, **z**^*SN*^)). Sometimes these differences are marginal, but other times they are significant. In Fig. 8, we show NMI(**z**^*Full*^, **z**^*SN*^) and the under segmentation error in panels A. and B., respectively. Defining super nodes with Slashburn has high NMI for both the Louvain algorithm and the SBM on the Amazon and Stanford networks. The maximum mean NMI(**z**^*Full*^, **z**^*SN*^) for Amazon and Stanford across various values of super nodes tested with the Louvain algorithm are 0.63 and 0.62, respectively. Using our core HD technique to define seeds and applying the Louvain algorithm gives NMI values of 0.69 and 0.62 for Amazon and Stanford, respectively. Similarly, the maximum mean NMI(**z**^*Full*^, **z**^*SN*^) for Amazon and Stanford networks with Slashburn under the SBM are 0.63 and 0.53, respectively. This compares to our results with CoreHD yielding maximum mean NMI(**z**^*Full*^, **z**^*SN*^) of 0.67 and 0.6 for the Amazon and Stanford networks, respectively. In the As22 networks, Slashburn produces maximum mean NMI(**z**^*Full*^, **z**^*SN*^) of 0.26 and 0.25 under the Louvain algorithm and SBM, respectively. Our results with Core HD are much stronger with maximum mean NMIs under Louvain and SBM of 0.56 and 0.46, respectively. These results can be more easily visualized in the Slashburn columns of Tables 2 and 3. We also observe larger variability in under segmentation error when defining super nodes with Slashburn.

**Table 2:** NMI(**z**^*Full*^, **z**^*SN*^) under the Louvain algorithm for *S* = 600 super nodes.

	CoreHD	Slashburn	KCBC
CMatter	**0.24**	0.17	0.09
As22	**0.56**	0.26	0.21
Enron	**0.39**	0.29	0.27
BrightKite	**0.31**	0.25	0.22
Amazon	**0.69**	0.63	0.35
Dblp	**0.23**	0.16	0.15
Email	**0.46**	0.34	0.31
Stanford	**0.62**	**0**.**62**	0.5
NotreDame	**0.5**	0.42	0.38

**Table 3:** NMI(**z**^*Full*^, **z**^*SN*^) under the SBM for *S* = 600 super nodes.

	CoreHD	Slashburn	KCBC
CMatter	**0.45**	0.39	0.25
As22	**0.46**	0.25	0.22
Enron	**0.37**	0.25	0.22
BrightKite	**0.23**	0.19	0.17
Amazon	**0.67**	0.63	0.39
Dblp	**0.41**	0.30	0.23
Email	**0.18**	0.12	0.15
Stanford	**0.6**	0.53	0.55
NotreDame	**0.52**	0.37	0.43

**Figure 9:**
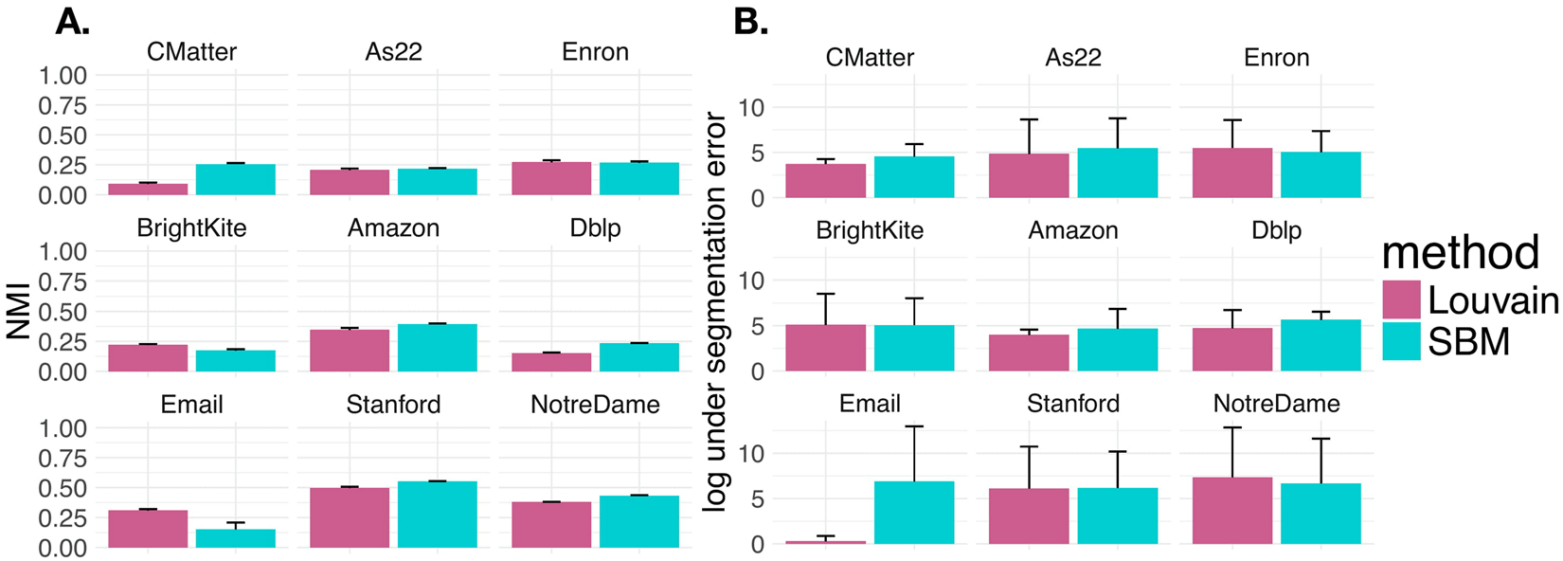
Super Node Quality with KCBC. Seeds were defined with KCBC. Since KCBC does not allow for the specification of the number of super nodes, we obtained only one super node representation result (hence the barplot). The results here show the super node quality results from Fig. 3, profiling profiling (NMI(**z**^*Full*^, **z**^*SN*^)) (**A**) and under segmentation error (**B**). We defined the super node representation 5 different times under this method, with error bars denoting standard deviation. While the NMI(**z**^*Full*^, **z**^*SN*^) are comparable to both CoreHD and slashburn, they are often inferior. The results for under segmentation error are comparable.

In Fig. 9 we show the results for (NMI(**z**^*Full*^, **z**^*SN*^)) and under segmentation error for the super nodes defined with KCBC. Again, under this method the number of super nodes is not specified and instead is determined based on the number of structures identified through the *k*-core based graph decomposition. Hence, for each network, we can not study how (NMI(**z**^*Full*^, **z**^*SN*^)) and under segmentation error vary as a function of the number of super nodes. Instead, we defined super nodes 5 times according to KCBC and created bar plots for our two super node quality metrics in each of the 9 networks under the Louvain algorithm and the SBM. In these plots, error bars denote standard deviation. In general, the KCBC approach does not perform as well as CoreHD and Slashburn. Defining super nodes with KCBC results in the strongest NMI(**z**^*Full*^, **z**^*SN*^) in the Stanford network under both the Louvain algorithm and SBM with values of 0.5 and 0.55, respectively. However, on this network both CoreHD and Slashburn yield superior results. Under the Louvain algorithm the maximum mean NMI of CoreHD and Slashburn are 0.62. With the SBM the maximum mean NMI values are 0.6 and 0.53, respectively. Similar to the pattern we observed in Fig. 8, KCBC also does not perform very well on NMI(**z**^*Full*^, **z**^*SN*^) in the As22 network. We refer to the KCBC column in Tables 2 and 3 for a closer look at these results. Under segmentation error is comparable to the CoreHD and Slashburn results.

We summarize the differences in the super node network performance obtained with CoreHD as opposed to Slashburn or KCBC by looking closely at the maximum NMI(**z**^*Full*^, **z**^*SN*^) obtained across all possible numbers of super nodes. Creating a super node representation of the network with *S* = 600 leads to the maximum value of NMI(**z**^*Full*^, **z**^*SN*^) among all values of *S* we considered in our experiments. We report the NMI(**z**^*Full*^, **z**^*SN*^) of all 9 networks under the Louvain algorithm and the SBM for a representation with *S* = 600 super nodes in Tables 2 and 3, respectively. Given the differences among the three compared methods for defining seeds (CoreHD, Slashburn, KCBC), it is important in future work to understand the properties of particular networks that lead to differences in performance among these methods.

## Discussion

We developed an approach for compressing a network into a super node representation that can be input to standard community detection algorithms. Using the smaller super node network reduces runtime and the variability between multiple runs of the same community detection algorithm. Our results also demonstrate that the communities in the super node network are better aligned with local network neighborhoods in a predictive sense, while still being in relatively good alignment with the partitions obtained using the full network.

Super nodes may be useful in a variety of contexts where large datasets are otherwise difficult to mine and interpret. For example, one might visualize the super node version of the network rather than the entire network, or use the members of a super node to identify redundant information in the network. Future work on super node representations can include the extension of this method to directed, signed, attributed, or bipartite networks. Additionally, one might consider a probabilistic model framework, attempting to infer latent super node assignments. Future work may also examine graph theoretic properties of super node representations in terms of how it aligns with the original network.

## Methods

We create a super node representation through three steps, outlined in Fig. 1. First, we define seeds. Next, we ‘grow’ super nodes by assigning the remaining nodes to seeds. Finally, the network of super nodes is defined by agglomerating edges, and can then be used in a community detection algorithm.

### Identify Seeds in the Network

To define *S* seeds, we aim to identify a set of nodes S* that satisfy the following criteria. First, seeds should have high centrality, or importance in the network. This ensures that we do not define a seed as some node on the periphery of the community and not similar to many other nodes in the network. Second, seeds should be effective at collapsing the network. Since our motivation for defining seeds is to serve as a center to agglomerate the remaining nodes around, each seed should be within close neighborhood regions for many nodes. Third, seeds should be well separated from one another so as to maximize the resulting compression. The most naive approach is to select nodes with highest degree, and this might be perfectly reasonable under various circumstances. Importantly, the selection of nodes with highest degree is computationally fast, requiring *O*(*M*) operations, summing over the *M* edges to calculate the degrees of the *N* nodes.

At slightly higher computational cost, we employ the CoreHD algorithm, which nearly optimally identifies nodes in network decycling and dismantling^28^. CoreHD recursively identifies the highest degree node in the 2-core. The 2-core is simply the maximal connected subgraph in which all nodes have at least degree 2. At each iteration, we add the identified highest degree node to our seed set and remove it from the network used by further CoreHD iterations. After the removal of this node, the 2-core is recomputed. The difference between selecting highest degree nodes and CoreHD for our present task may be small, both in terms of result and computational cost. In particular, because we will only select $S\ll N$ seeds, there is reduced opportunity for the removals to lead to subgraphs with substantial differences between degree order in the graph and its 2-core. Indeed, in our experience, simply selecting the highest degree nodes as the seeds often works well in practice. Because of the minimal extra computational cost for computing the 2-core, we use CoreHD for all of our results shown here.

We were motivated to use the 2-core based on the results in ref.^28^, where in the context of the network decycling and dismantling problem, updating the 2-core in each iteration after deleting high degree nodes is convenient and can quickly decompose the network into smaller components.

### Grow Super Nodes Around Seeds

Once the set of seeds, S*, is defined, we ‘grow’ them out agglomerating nearby nodes to build the super nodes. We formally define a super node as a subset of one or more nodes from the original network, ${\mathscr{X}}$. To do this, seeds are grown out to engulf nodes in increasing neighborhood orders until either all nodes are assigned to a super node center or until a user-defined number of neighborhood orders has been considered. The maximum order, *o*_*max*_, can be specified to control the maximum order neighborhood to consider in building the super nodes. If after *o*_*max*_, there are still unassigned nodes, the unassigned nodes are not used to build the new super node network and are all ultimately assigned to the same periphery community as they are not considered relevant to the network core. Depending on the number of chosen super nodes, *S*, the degree distribution of the original network and the quality of the chosen seeds at collapsing the network, different networks will require repeating the agglomeration process for different neighborhood orders if one wants to ensure every node is assigned to a super node. In all of our experiments in this paper, we used *o*_*max*_ = 6, as we observed that for most networks this value successfully assigns the majority of the nodes to a super node. The output is a vector, **s** of length *N*, which gives the node-to-super-node assignments for the nodes in the original network.

### Create Network of Super Nodes

Finally, after growing the super nodes, we create a new network representation of the super nodes. To do this, we create a weighted network, ${\mathscr{W}}$, where each super node is a node and the weight of the edge between a pair of distinct super nodes is the total weight of edges in the original network ${\mathscr{X}}$ between pairs of nodes assigned to the respective super nodes. For pairs of super nodes whose members have no edges between them in ${\mathscr{X}}$, there is no corresponding edge in ${\mathscr{W}}$. By definition, we construct ${\mathscr{W}}$ with no self loops. Moreover, the produced super node network representation produces a weighted network where the edge weights are counts.

After applying community detection to the super nodes, their community assignments are mapped back to their constituent *N* nodes of the original network, ${\mathscr{X}}$. We denote this final *N*-length matched community assignment as **z**. In experiments in the Results section, we consider the node-to-community assignment **z**^*Full*^ obtained by applying community detection to the full network and the mapped result **z**^*SN*^ obtained by applying community detection to the super node representation.

### Code availability

Our code for creating a super node representation of a network is available on github: https://github.com/stanleyn/SuperNode.
